# Systemic Metabolic Changes in Plasma of Patients with Myelodysplastic Neoplasms and Chronic Myelomonocytic Leukemia

**DOI:** 10.3390/metabo16090648

**Published:** 2026-09-04

**Authors:** Ekaterina Balaian, Iryna Kovtun, Fabian Springer, Denise Medeiros Selegato, Sophie Jonas, Uta Oelschlaegel, Manja Wobus, Michael Wulfert, Corinna Strupp, Ulrich Germing, Michael Zimmermann, Martin Bornhäuser, Triantafyllos Chavakis, Katja Sockel, Alexander Funk

**Affiliations:** 1 Department of Internal Medicine I, University Hospital Carl Gustav Carus Dresden, Dresden University of Technology, 01307 Dresden, Germany; iryna.kovtun@ukdd.de (I.K.);; 2 German Cancer Consortium (DKTK), Partner Site Dresden and German Cancer Research Center (DKFZ), 69120 Heidelberg, Germany; 3 National Center for Tumor Diseases (NCT/UCC), 01307 Dresden, Germany; 4 Molecular Systems Biology Unit, European Molecular Biology Laboratory (EMBL), 69117 Heidelberg, Germany; 5 Institute for Clinical Chemistry and Laboratory Medicine, University Hospital Carl Gustav Carus, Dresden University of Technology, 01307 Dresden, Germany; 6 Department of Hematology, Oncology, and Clinical Immunology, Heinrich-Heine University, 40225 Dusseldorf, Germany

**Keywords:** myelodysplastic neoplasms, MDS, CMML, metabolism, lipids

## Abstract

Background: Myelodysplastic neoplasms (MDSs) are clonal hematopoietic stem cell disorders associated with ineffective hematopoiesis, chronic inflammation, and increased cardiovascular morbidity. Although metabolic dysregulation has been implicated in MDS pathogenesis, systemic metabolic alterations remain incompletely characterized. Methods: Plasma samples from treatment-naïve patients with MDS or chronic myelomonocytic leukemia (CMML) and age-matched healthy controls were analyzed using quantitative nuclear magnetic resonance spectroscopy and liquid chromatography-mass spectrometry (LC-MS). Metabolomic profiles were compared using unsupervised and supervised multivariate analyses, validated in an independent external MDS cohort, and integrated with re-analysis of publicly available RNA-sequencing datasets from purified CD14^+^ CMML monocytes. Results: Patients with MDS and CMML exhibited broad reductions in circulating lipoprotein-associated metabolites, including HDL-, LDL-, IDL-, and apolipoprotein-associated fractions, indicating disturbed systemic lipoprotein homeostasis. Within the discovery cohort, CMML samples showed higher concentrations of the ketone bodies 3-hydroxybutyrate and acetoacetate, as well as succinate. LC-MS analysis demonstrated selective increases in C18:1 acylcarnitine, oleic and isopalmitic acids, whereas free carnitine abundance remained unchanged. Elevated 3-hydroxybutyrate levels were not associated with mutational burden, hematologic parameters, disease risk, or immunophenotypic features. Re-analysis of public CMML monocyte transcriptomes demonstrated increased expression of genes involved in lipid uptake and intracellular lipid trafficking, including *FABP5*, *APOE*, *LPL*, and *SLC27A2*, without coordinated activation of fatty acid oxidation pathways. External cohort analysis confirmed the overall MDS-associated plasma metabolomic profile. Conclusions: MDSs and CMML are associated with reproducible alterations in systemic lipid metabolism characterized by reduced circulating lipoprotein-associated metabolites, while CMML showed more pronounced ketone body- and acylcarnitine-associated metabolic phenotype accompanied by changes in lipid-handling transcriptional programs. These findings support altered systemic lipid metabolism and carnitine-dependent fatty acid handling as characteristic features of myeloid neoplasms and provide a rationale for future functional studies investigating lipid metabolism in disease pathogenesis.

## 1. Introduction

Myelodysplastic neoplasms (MDSs) are a group of clonal hematopoietic stem cell (HSC) malignancies characterized by ineffective hematopoiesis, bone marrow (BM) dysplasia, persistent peripheral blood (pB) cytopenias and an increased risk of progression to acute myeloid leukemia (AML). At the molecular level, MDSs are driven by recurrent mutations that can profoundly influence HSC function and are accompanied by chronic BM inflammation, oxidative stress, and dysregulated immune signaling. MDS are predominantly diseases of the elderly, with a median age at diagnosis of approximately 70–75 years and an increasing incidence with advancing age [1].

Although MDSs are defined by ineffective hematopoiesis, it is increasingly recognized as a systemic disease in elderly, comorbid population, in which non-leukemic events are a major determinant of survival. Autoimmune and inflammatory conditions occur in approximately 10–30% of MDS patients and include vasculitis, connective tissue disorders, and inflammatory arthritis [2,3]. In addition, numerous studies have demonstrated that patients with MDSs exhibit a markedly increased burden of cardiovascular comorbidities, including ischemic heart disease, heart failure, stroke, and thromboembolic events [4,5]. Cardiovascular disease (CVD) represents a leading cause of non-leukemic mortality in MDS, particularly among patients with lower-risk disease [6,7]. Epidemiological findings also link MDS, clonal hematopoiesis and cardiovascular pathology. Chronic, low-grade inflammation is a hallmark of MDSs and clonal hematopoiesis, characterized by elevated levels of pro-inflammatory cytokines such as TNF-a, IL-6 and IL1-β. These inflammatory mediators are well-established drivers of most frequent CVD [8], providing a biological rationale for the increased cardiovascular morbidity observed in MDS patients. In parallel, clonal hematopoiesis alone has emerged as an independent risk factor for CVD [9,10]. These observations support the concept that MDS should be considered as a multisystem disorder, in which malignant hematopoiesis interacts with systemic inflammation, immune dysfunction, and metabolic imbalance.

Cellular metabolism can be a plausible biological bridge between clonal hematopoiesis, chronic inflammation and systemic complications in MDS. Metabolic pathways govern HSC quiescence and differentiation, expansion of immune cells, and contribute to vascular homeostasis [11]. Perturbations in glycolysis, mitochondrial function, fatty acid oxidation, and amino acid metabolism can simultaneously influence clonal fitness, inflammatory state, and endothelial dysfunction.

Thus far, only a limited number of studies have demonstrated specific metabolic changes in MDS patients, including alterations in amino acid and purine metabolism, lipid and eicosanoid pathways, oxidative stress-related metabolites, and central energy metabolism [12,13], suggesting broad dysregulation of redox balance, mitochondrial function, and inflammatory signaling.

However, findings remain partially inconsistent, with some reports indicating enhanced mitochondrial activity [14], while others documented impaired mitochondrial function [15,16,17], potentially reflecting differences across cell type, disease stages, risk groups, and genetic backgrounds.

Despite increasing interest in this area, the metabolic alterations associated with MDSs remain incompletely understood. Therefore, in this study, we aimed to conduct a comprehensive analysis of plasma metabolites in MDS patients and age-matched controls to identify possible disturbances associated with the clinical manifestation of MDSs.

## 2. Methods

### 2.1. Patients

Blood plasma samples from 14 healthy donors (median age at sampling 60 years (range, 54–70; m/f = 6/8)) and 30 treatment-naïve patients with MDS (n = 22) or CMML (n = 8) at the University Hospital Dresden (patients, median age at sampling 66 years (range, 41–81; m/f = 24/6)) were prospectively collected and stored at −80 °C upon centrifugation within a maximum of 4 h. Donors were considered healthy if they had no known hematological or oncological disorders. Demographic and clinical characteristics of the discovery cohort are summarized in Table 1.

MDS/CMML was diagnosed based on morphological analysis of BM smears by two independent hematologist experts, as well as available cytogenetical and molecular data. Of these patients, 15 presented with lower-risk MDS/CMML (very low, low, and intermediate according to Revised International Prognostic Scoring System (IPSS-R); low and intermediate-1 according to CPSS-Mol) and 15 had high risk MDS/CMML (high and very high according to IPSS-R; intermediate-2 and high according to CPSS-Mol). The median number of MDS-specific mutations was 3 (range, 1–10, information not available for one patient); TP53 mutation was detected in 5 patients. pB counts at the time of sampling were as follows: hemoglobin concentration 6.6 ± 1.5 mmol/L vs. 8.24 ± 0.96 mmol/L, leukocytes 3.61 ± 2.48 GPt/L (MDS), 15.89 ± 9.60 GPt/L (CMML) vs. 7.05 ± 2.71 GPt/L, and lymphocytes 1.61 ± 1.06 GPt/L vs. 2.09 ± 0.87 GPt/L, in MDS and healthy, correspondingly. Concentration of erythropoietin has been assessed in MDS patients as a routine parameter and comprised 40.0 IU/mL (range, 9.2–749.9 IU/mL, reference range 5.4–31.0 IU/mL). Time to plasma sampling from the diagnosis varied from 1 to 205 months (median 11.5 months).

Cardiovascular comorbidities (ischemic heart disease, arterial hypertension, and peripheral arterial disease) were present in 67% of MDS/CMML patients and in 57% of healthy counterparts. Five MDS/CMML patients (17%) and four healthy counterparts (29%) used hypolipidemic drugs as regular medication.

Forty-nine plasma samples from treatment-naïve MDS patients for the validation cohort were provided by the MDS-Biobank at University Hospital Düsseldorf (median age at sampling 69 years, range 40–91). Cardiovascular comorbidities were present in 27 out of 49 patients (55%). Time from diagnosis to plasma sampling ranged from 0 to 11 months. Three patients received hypolipidemic drugs at the time of sampling, whereas comedication data were unavailable for 26 patients (53%) due to the retrospective nature of data acquisition. The number of MDS-specific mutations ranged from 0 to 5 (median 1); molecular data were unavailable for 22 patients (45%). According to IPSS-R, 30 patients were classified as lower risk and 15 as higher risk; data were unavailable for four patients. Erythropoietin concentration was 200.7 IU/mL (range 4–2118 IU/mL), available for 34 patients (69%). pB counts were as follows: hemoglobin 6.21 mmol/L (range 3.35–9.56 mmol/L), leukocytes 3.59 GPt/L (range 0.9–28.3 GPt/L), lymphocytes 1.2 GPt/L (range 0.4–4.2 GPt/L), and platelets 111 GPt/L (range 3–576 GPt/L).

This study was conducted within the framework of the German MDS Registry, approved by the local ethics committees in Dresden and Düsseldorf, and carried out in accordance with the Declaration of Helsinki. Written informed consent was obtained from all participants.

### 2.2. Metabolomics

^1^H NMR spectroscopy measurements were performed according to established protocols [18,19]. The frozen plasma samples were thawed at room temperature for about 30 min before being mixed with phosphate buffer (1:1) to a volume of 600 µL. The phosphate buffer also contained the internal reference TMSP. The resulting mixture was pipetted into the NMR sample tube and immediately prepared for measurement.

All NMR measurements were run on a Bruker 600 Mhz Avance III Neo (Bruker BioSpin GmbH & Co. KG, Ettlingen, Germany) equipped with a BBI Probe and a Bruker SampleJet robot with a cooling system for sample storage at 4 °C. The samples were measured at 37 °C and a full quantitative calibration was completed before the measurement. All measurements followed the Bruker in vitro diagnostics (IVDr) SOPs and methods. All data were processed in automation using Bruker TopSpin 4.5.0 and ICON NMR. Automatic metabolite and lipoprotein reports were obtained using Bruker IVDr B.I Methods Plasma (B.I.Quant-PS, v2.5.0) and Bruker IVDr Lipoprotein Subclass Analysis (B.I.LISA, v1.1.0). The analysis was performed using a 1D Nuclear Overhauser Effect Spectroscopy experiment (Bruker BioSpin GmbH & Co. KG, Ettlingen, Germany).

### 2.3. Liquid Chromatography-Mass Spectrometry (LC-MS)

#### 2.3.1. Sample Preparation

Blood samples were prepared for LC-MS analysis by organic solvent extraction. In brief, 20 μL of supernatant was mixed with 105 µL of organic solvent (acetonitrile:methanol, 1:1) supplemented with an internal standard mixture. Internal standard mixture consisted of phenylalanine-d5, tryptophan-d5, ibuprofen-d4, tolfenamic acid-d4, estriol-d3, diclofenac-d4, warfarin-d5, oxfendazole-d3, chloramphenicol-d5, nafcillin-d5 and caffeine-d9, each to a final concentration of 800 nM. The material was homogenized and incubated at −20 °C to allow protein precipitation. After incubation, samples were centrifuged (3220× *g*, 4 °C) for 15 min. For C18 LC-MS acquisition, 15 µL of supernatant was diluted with 30 µL H_2_O.

#### 2.3.2. LC-MS Acquisition

LC-MS analysis was performed using reverse-phase chromatography in positive and negative ionization mode. Chromatographic separation was performed using an Agilent 1200 Infinity UHPLC system with an InfinityLab Poroshell HPH-C18 column (2.1 × 100 mm, 1.9 µm particle size) operated at 45 °C (Agilent Technologies, Santa Clara, CA, USA). Mobile phases were water with 0.1% formic acid (solvent A) and methanol with 0.1% formic acid (solvent B). Five microliters of the sample were injected with a gradient from 5% to 95% B over 6.5 min.

The qTOF instrument (Agilent 6550) was operated in scanning mode (50–1700 *m*/*z*) at the following source parameters: VCap: 3500 V, nozzle voltage: 2000 V, gas temp: 225 °C; drying gas 13 L/min; nebulizer: 20 psig; sheath gas temp 225 °C; sheath gas flow 12 L/min. Online mass calibration was used throughout the run in a second ionization source and a constant flow of reference ion solution (*m*/*z* 922.010 and 121.051 for positive mode and *m*/*z* 112.9857 and 1033.9881 for negative).

Tandem mass spectrometry analysis (LC-MS/MS) was performed for all metabolites using the chromatographic separation and source parameters described above and the targeted-MS/MS mode of the instrument with a preferred inclusion list for parent ion with 20 ppm tolerance, Iso width set to ‘narrow width’, and collision energy set to either 10, 20, or 40 eV.

#### 2.3.3. Validation of the Untargeted Metabolomics Results

The area under the curve from significant features was integrated using the MassHunter Quantitative Analysis Software (Agilent, version 7.0, Agilent Technologies, Santa Clara, CA, USA) based on the accurate high-resolution mass and RT of the reference analytes and the following parameters: signal threshold of 30,000; mass tolerances of 0.002 amu or 20 ppm; retention time tolerance of 0.2 min.

Lastly, all metabolites annotated in this manuscript were assigned a Level 2 annotation according to the Metabolomics Standards Initiative (MSI) classification system. This includes a match in retention time using the same chromatographic system and accurate mass.

#### 2.3.4. Statistical Analysis of LC-MS Metabolomics

Raw peak intensities were normalized to the mode-specific internal standard, using chloramphenicol-d5 for C18 negative-ion mode and caffeine-d9 for C18 positive-ion mode. For each sample, the analyte intensity was divided by the internal-standard signal relative to its median across all samples. Raw analyte peak intensities below the signal threshold of 30,000, including non-integrated peaks, were treated as non-detected and set to zero before normalization. After normalization, a pseudocount of 1000 was added and intensities were log2-transformed. Differential metabolite abundances between groups were assessed using pairwise two-sided Wilcoxon rank-sum tests.

### 2.4. Flow Cytometry

Subpopulations of T lymphocytes and NK cells were analyzed using flow cytometry. The subpopulations were characterized as described by Koch et al. [20] and Tentori et al. [21], correspondingly. The 8-color-antibody panel is summarized in Supplementary Table S1. A lyse–stain–wash procedure was performed. At least 200.000 CD45-positive leukocytes were recorded using a FACS-Canto10 flow cytometer (BD Biosciences, San Jose, CA, USA) equipped with three lasers.

### 2.5. Analysis of Publicly Available CMML CD14^+^ Monocyte RNA-Seq Datasets

Publicly available RNA-seq data from purified CD14^+^ monocytes were obtained from GEO datasets GSE135902 and GSE283203 using the GEOquery (version 2.80.0) [22] in R (version 4.6.1), and samples were classified as CMML or healthy controls based on GEO sample annotations. Ensembl identifiers were mapped to official gene symbols using AnnotationDbi (version 1.74.0) and org.Hs.eg.db (version 3.23.1), and duplicated gene symbols were collapsed by summing counts. Lowly expressed genes with total counts ≤10 across all samples were excluded. Differential expression analysis of CMML versus healthy CD14^+^ monocytes was performed using DESeq2 (version 1.52.0) [23], with Benjamini–Hochberg correction for multiple testing. Selected genes related to fatty acid uptake, carnitine transport, fatty acid oxidation, and ketone body metabolism were visualized using ggplot2 (version 4.0.3) and pheatmap (version 1.0.13).

### 2.6. Use of AI-Assisted Language Editing

AI-assisted language editing was performed using ChatGPT (OpenAI, GPT-5.5) to improve the grammar, readability, and structure of the manuscript. AI tools were not used for experimental design, data generation, statistical analysis, or interpretation of the results. All scientific content and the final manuscript text were reviewed and approved by the authors.

### 2.7. Statistics

Statistical analyses were performed using MetaboAnalyst 6.0 [24] and R (version 4.4.1). Parameters with more than 50% missing values were excluded. Glucose and lactate were excluded because of uncertain pre-analytical sample handling. Remaining missing values were imputed with one-fifth of the minimum observed value for each metabolite before log transformation. Data were natural log-transformed and mean-centered prior to multivariate analyses. Group comparisons were performed using the Wilcoxon rank-sum test, and correlations were assessed using Spearman’s rank correlation coefficient. To assess whether differences in sex distribution or lipid-lowering medication use influenced the main findings, additional analyses were performed for selected key metabolites. Multivariable regression models were used to compare the disease groups while adjusting for sex and lipid-lowering medication use. Metabolite concentrations were log-transformed before analysis, and *p* values were corrected for multiple testing using the Benjamini–Hochberg false discovery rate (FDR) method. For heatmap visualization, metabolite abundances were Z-score normalized for each metabolite. PLS-DA was performed using MetaboAnalyst 6.0. Model performance was assessed by R^2^, Q^2^ obtained by cross-validation, classification accuracy, balanced accuracy, and area under the ROC curve (AUC). Statistical significance of the PLS-DA model was evaluated by 1000 permutation tests. Unless otherwise specified, all statistical tests were two-sided and *p* < 0.05 was considered statistically significant.

## 3. Results

### 3.1. Broad Suppression of Circulating Lipoprotein-Associated Metabolites in MDS

**Figure 1:**
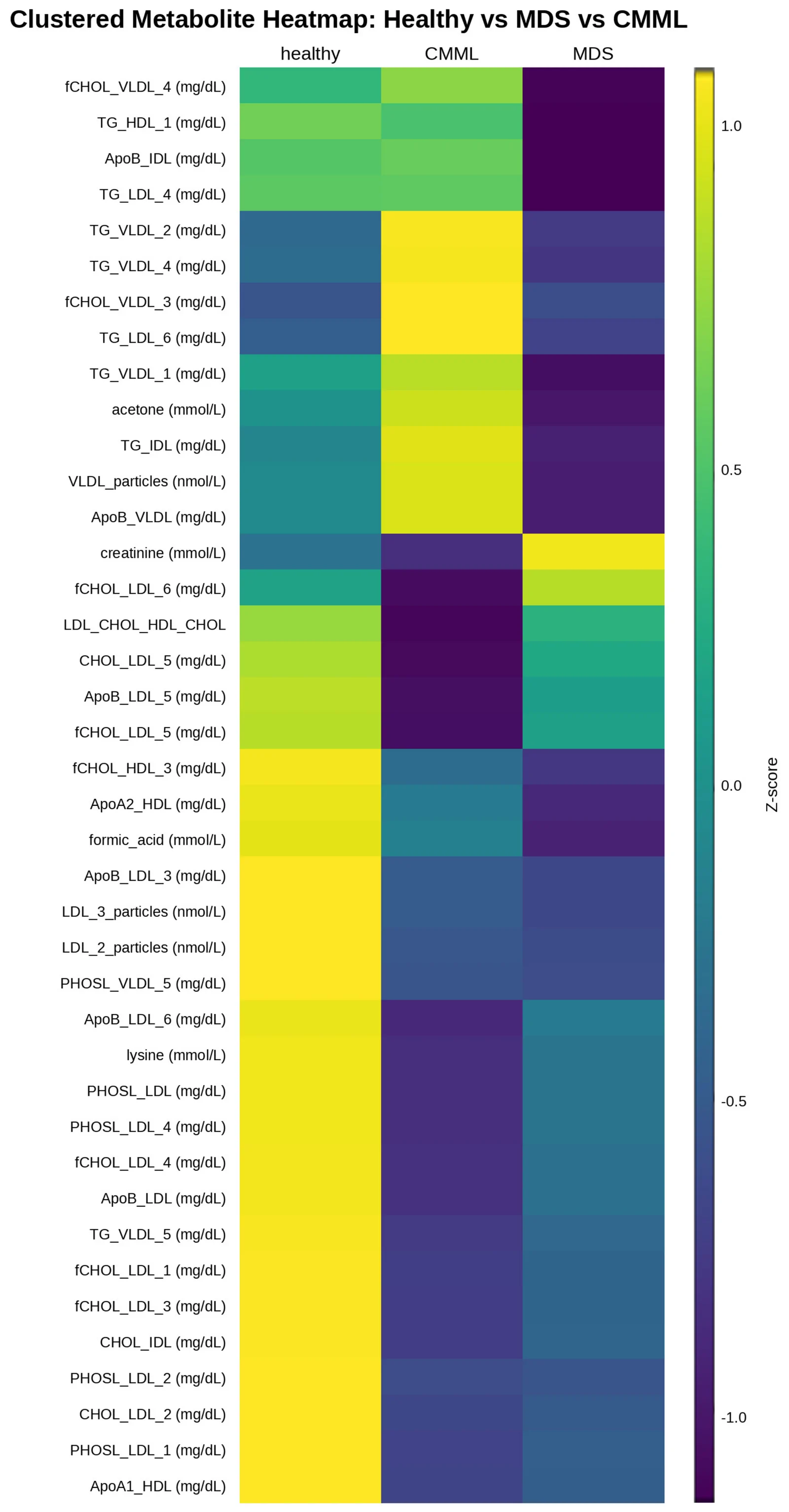
Clustered heatmap of plasma metabolomic profiles in healthy controls (n = 14), MDS (n = 22), and CMML (n = 8) patients. Heatmap shows relative metabolite abundance in plasma across healthy controls and patients with MDS or CMML. Yellow indicates relatively higher abundance, whereas purple indicates relatively lower abundance. The heatmap demonstrates distinct metabolomic signatures between healthy individuals and patients with myeloid neoplasms, with multiple lipid- and lipoprotein-associated metabolites enriched in healthy controls and reduced in MDS/CMML samples.

To explore global metabolic differences between healthy controls and patients with MDS/CMML, we performed hierarchical clustering of metabolomic profiles obtained by NMR spectroscopy (Figure 1). Z-score-normalized metabolite abundances revealed distinct disease-associated metabolic patterns across the three groups.

Compared with healthy controls, patients with MDS and CMML demonstrated broad alterations in lipid- and lipoprotein-associated metabolites, with reduced levels of multiple HDL-, LDL-, and apolipoprotein-associated metabolites. These included decreased apolipoproteins A-I/-II in high-density lipoproteins (HDLs), apolipoprotein B in low- (LDLs) and intermediate-density lipoproteins (IDLs), total cholesterol in LDL subfraction 1 and 2, as well as free cholesterol in HDL subfraction 3, and several phospholipid-containing LDL fractions, including phospholipids in LDL subfraction 1, 2 and 4 (Figure 1). These findings demonstrate broad quantitative alterations in circulating lipoprotein-associated metabolites in both myeloid neoplasms but do not distinguish between changes in lipoprotein production, utilization, remodeling, or clearance.

Direct comparison between the disease groups identified additional alterations predominantly associated with CMML with relative enrichment and preservation of several triglyceride- and VLDL-associated metabolites, including triglycerides in VLDL subfraction 1, 2, 4, and 5, as well as VLDL particle concentration (Figure 1). In addition, differential clustering of LDL particle subclasses, particularly LDL subfraction 2 and 3 particle concentration, further distinguished CMML from MDS, suggesting disease-specific alterations in lipoprotein particle composition and lipid handling. Collectively, these findings support the presence of shared lipid metabolic dysregulation across both patient groups, accompanied by additional differences in triglyceride- and lipoprotein-associated metabolite profiles that were most pronounced in CMML.

Beyond lipid-associated metabolites, amino acid metabolism was also altered. In particular, lysine levels were reduced in MDS samples compared with healthy controls, suggesting that non-lipid metabolic pathways may also contribute to disease-associated metabolic remodeling.

### 3.2. 3-Hydroxybutyrate Is Increased in Patients with MDS/CMML

**Figure 2:**
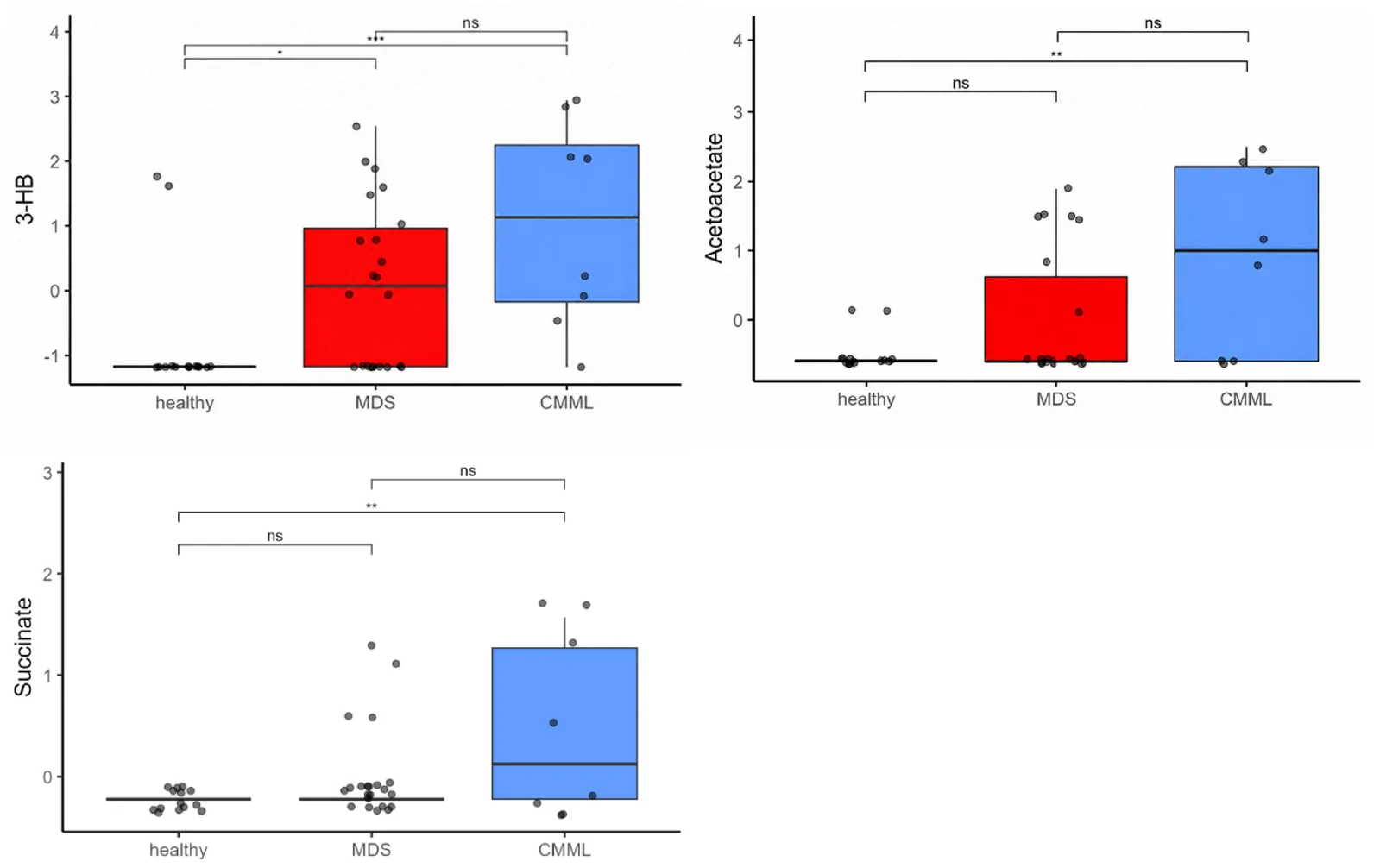
Concentration of 3-hydroxybutyrate (3-HB), acetoacetate, and succinate in healthy donors (n = 14) and MDS (n = 22) and CMML (n = 8) patients. Concentrations were measured in mmol/L. For statistical analysis and visualization, values were log-transformed and mean-centered. Effect sizes for CMML versus healthy controls were estimated using rank-biserial correlations (3-HB: r_rb_ = 0.75; acetoacetate: r_rb_ = 0.57; succinate: r_rb_ = 0.50).* *p* < 0.05, ** *p* < 0.01, *** *p* < 0.001, ns—not significant.

To further characterize disease-associated metabolic alterations, we analyzed ketone body- and lipid-associated metabolites in patients with MDS/CMML and in healthy controls. Ketone body-associated alterations were most pronounced in CMML, including 3-hydroxybutyrate (3-HB) (0.219 mmol/L in CMML vs. 0.025 mmol/L in healthy controls; *p* = 0.0015), whereas 3-HB levels in MDS patients were intermediate (0.072 mmol/L) and did not differ significantly from CMML. A similar pattern was observed for acetoacetate (CMML 0.041 vs. healthy 0.001 mmol/L; *p* = 0.0093; MDS 0.011 mmol/L), and succinate (CMML 0.011 vs. healthy 0.000 mmol/L; *p* = 0.0053; MDS 0.003 mmol/L) (Figure 2). In contrast, pyruvate, citrate, acetone, and 2-hydroxybutyrate did not show major differences between groups (data available on request).

Because healthy controls and patients differed in sex distribution and use of lipid-lowering medication, we performed additional multivariable sensitivity analysis adjusting for these potential confounders. The principal disease-associated metabolic alterations remained evident after adjustment. In particular, 3-HB, acetoacetate, and succinate remained significantly higher in CMML compared with healthy controls (*p* = 0.0051, *p* = 0.0138, and *p* = 0.0191, respectively). Similarly, major lipoprotein-associated alterations remained significantly different in both MDS and CMML compared to healthy controls. These associations also remained significant after FDR correction across the selected metabolite panel (Supplementary Table S2). Thus, differences in sex distribution and lipid-lowering medication use did not account for the principal metabolic alterations observed in the discovery cohort.

To further characterize alterations in fatty acid metabolism, plasma free carnitine, selected free fatty acids, and acylcarnitines were analyzed by LC–MS (Supplementary Figure S1). Free carnitine abundance did not differ significantly between healthy controls, patients with MDS, and patients with CMML. Among the acylcarnitines analyzed, C18:1-acylcarnitine abundance was significantly higher in CMML than in MDS, while no significant difference was observed between CMML and healthy controls. Oleic and isopalmitic acid abundances were significantly increased in CMML compared with both healthy controls and MDS, whereas MDS did not differ significantly from healthy controls (Supplementary Figure S1). Other acylcarnitines showed signal intensities close to the lower detection limit in the majority of samples and were therefore not considered for comparative analyses. Collectively, these findings demonstrate selective alterations in plasma acylcarnitine and fatty acid profiles in MDS and CMML, with the most pronounced changes observed in CMML.

**Figure 3:**
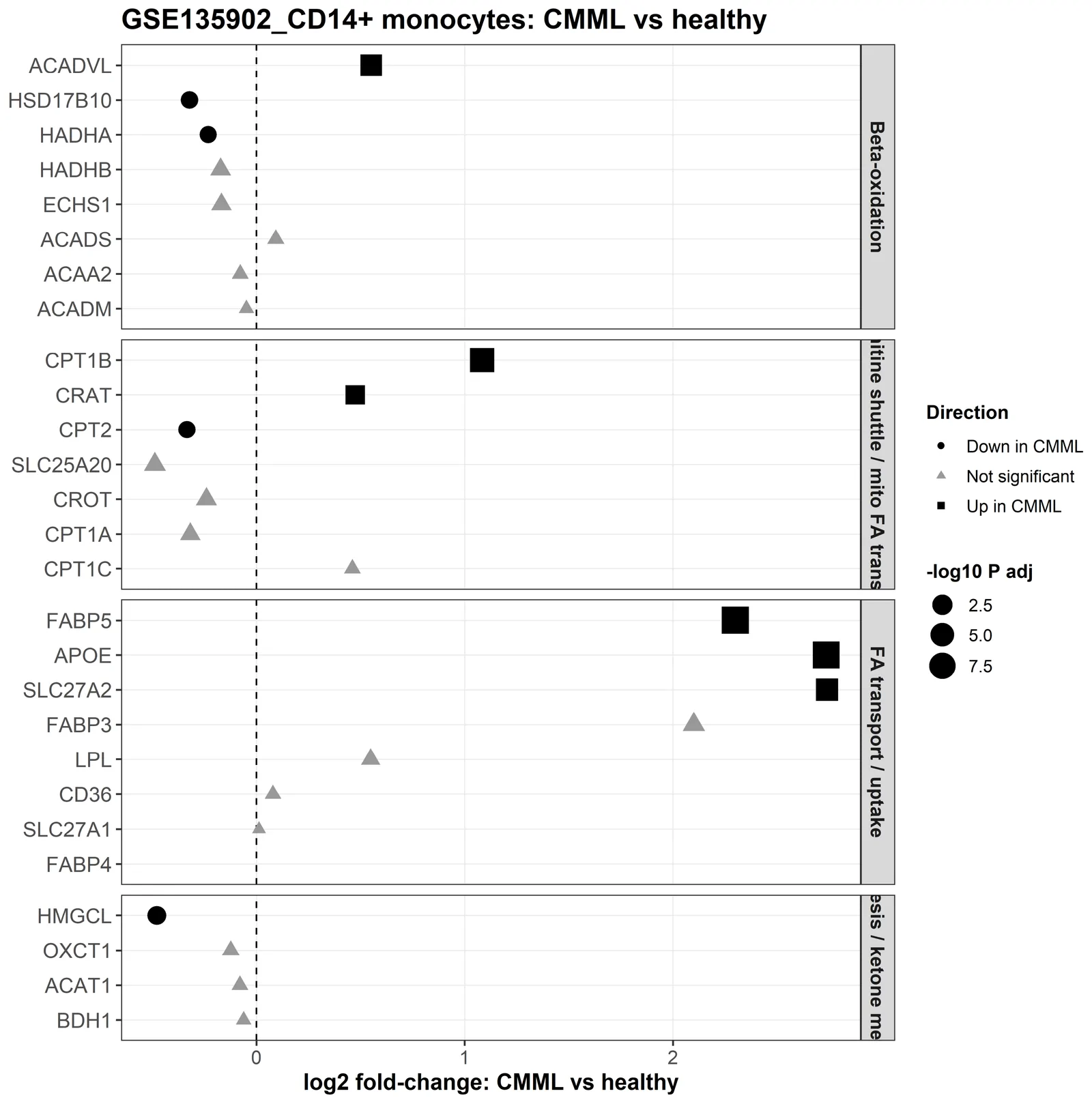
Lipid metabolism gene expression in CMML CD14^+^ monocytes. Dot plot showing differential expression of selected lipid metabolism-related genes in CMML versus healthy CD14^+^ monocytes from the published datasets. Genes are grouped by functional category, the *x*-axis shows log_2_ fold-change in CMML relative to healthy monocytes, and the dashed line indicates no change.

To explore whether these circulating metabolite changes were associated with altered lipid metabolic programs, we re-analyzed publicly available RNA-seq datasets of primary CD14^+^ CMML monocytes [25,26]. Across two independent cohorts, CMML monocytes consistently demonstrated increased expression of genes involved in fatty acid uptake and intracellular lipid trafficking, including *FABP5*, *APOE*, *LPL*, and *SLC27A2* (Figure 3). In contrast, coordinated induction of downstream mitochondrial fatty acid oxidation (FAO) and ketone-utilization pathways was not observed. While some FAO-associated genes showed partial upregulation, the overall transcriptional pattern was consistent with a gene-expression signature associated with enhanced lipid acquisition rather than uniform activation of complete oxidative fatty acid metabolism. However, these transcriptomic data do not demonstrate increased functional lipid uptake or intracellular lipid accumulation.

Together, these findings indicate shared alterations in systemic lipid metabolism across MDS and CMML, with ketone body- and selected fatty acid-associated changes being most pronounced in CMML. The combination of reduced circulating lipoprotein-associated metabolites in both disease groups, higher ketone body levels in CMML compared with healthy controls, and increased expression of lipid uptake-associated genes in CMML monocytes suggests additional metabolic remodeling in CMML. However, because ketone body levels did not differ significantly between MDS and CMML, these alterations should be considered CMML-enriched rather than CMML-specific.

### 3.3. Correlation Analysis of 3-HB with Clinical Data

We next assessed whether circulating 3-HB levels were associated with clinical or genetic features in the combined MDS/CMML patient cohort. No significant linear correlation was observed between 3-HB levels and mutational burden in the combined MDS/CMML cohort (Spearman ρ = 0.184, *p* = 0.257). However, individual patients with high mutational complexity demonstrated markedly elevated 3-HB concentrations, including cases with 10 mutations (3-HB: 0.25 mmol/L), 9 mutations (0.41 mmol/L), and 5 mutations (0.61 mmol/L). Conversely, other highly mutated cases showed undetectable 3-HB levels, indicating that increased 3-HB is not uniformly associated with mutational burden.

Interestingly, all patients harboring TP53 mutations (n = 5) exhibited non-measurable 3-HB levels, whereas TP53 wild-type patients showed variable 3-HB concentrations (Mann–Whitney U test, *p* = 0.025). Given the very small number of TP53-mutated cases, this observation should be regarded as exploratory and hypothesis-generating rather than evidence of a significant biological association.

In contrast, after FDR correction, no significant correlations were observed between 3-HB levels and other clinical parameters, including blast percentage, hemoglobin levels, leukocyte counts, erythropoietin levels, clinical risk groups, IPSS-R score, or immunophenotypic parameters. Collectively, these findings suggest that elevated 3-HB levels do not simply reflect global disease burden, hematologic severity, or mutational complexity, but may instead identify metabolically distinct patient subgroups within myeloid neoplasms.

### 3.4. PLS-DA Identifies Metabolomic Signature Separating Healthy Controls from MDS/CMML Patients

**Figure 4:**
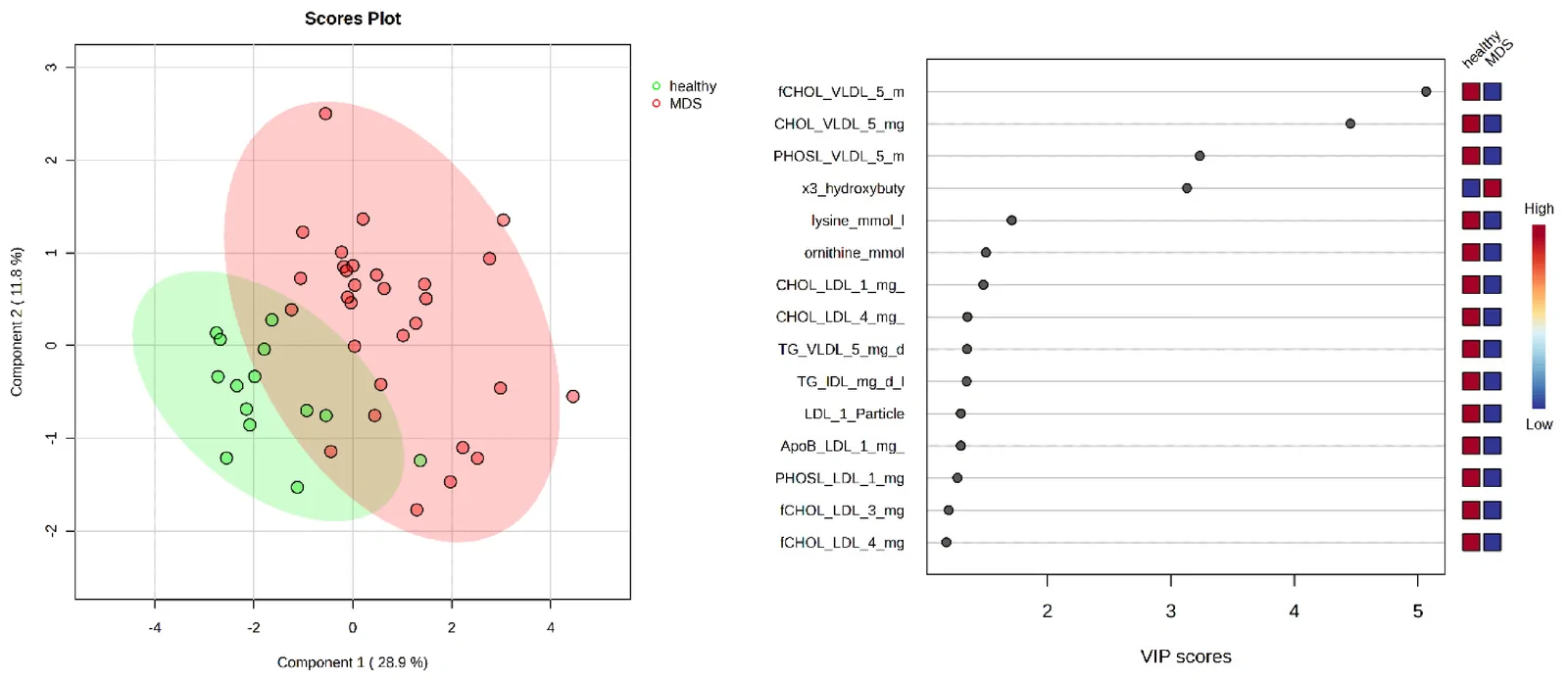
PLS-DA comparing MDS/CMML (red, n = 30) and healthy (green, n = 14) cohort reveals moderate but reproducible separation. The left panel shows the PLS-DA scores plot. The right panel displays the Variable Importance in Projection (VIP) scores, indicating the contribution of each metabolite to the discrimination between groups; higher VIP scores reflect a greater contribution to the model. The adjacent two-column heatmap shows the relative abundance of each metabolite in healthy controls and patients with MDS/CMML, with red indicating relatively higher and blue relatively lower levels according to the color scale. The gray dots represent VIP scores and are independent of the heatmap color coding. The performance of PLS-DA models with increasing numbers of latent components is shown in Supplementary Figure S2.

To assess whether the observed metabolomic differences could distinguish healthy controls from patients with myeloid neoplasms, we performed partial least squares discriminant analysis (PLS-DA) after exclusion of glucose- and lactate-related variables. MDS and CMML samples were analyzed together as a combined disease group, as the aim was to evaluate the shared metabolic signature of myeloid neoplasia rather than disease-specific differences. In addition, the small number of CMML samples (n = 8) precluded a robust separate multigroup PLS-DA analysis. PLS-DA demonstrated moderate but reproducible separation between healthy controls and MDS/CMML samples (Figure 4), with the strongest predictive performance observed using a single latent component (Supplementary Figure S2). Addition of further components increased the explained variance (R^2^) but did not improve predictive capacity, suggesting progressive overfitting.

PLS-DA permutation testing was performed to assess the robustness of the metabolomic separation between healthy controls and patients with MDS/CMML. The one-component PLS-DA model demonstrated good classification performance with an accuracy of 0.886, balanced accuracy of 0.840, and an area under the ROC curve (AUC) of 0.852. The corresponding cross-validated predictive ability was moderate, with a Q^2^ value of 0.324. Despite this moderate predictive capacity, the model consistently identified biologically coherent metabolite alterations, including reduced circulating lipoprotein-associated lipids and increased 3-HB.

To evaluate whether the observed separation could occur by chance, we performed 1000 permutation tests with randomized class labels. The observed Q^2^ value exceeded all permuted models, yielding an empirical *p* value of 0.001. Similarly, classification accuracy, balanced accuracy, and AUC were all significantly higher than expected by chance, with empirical *p* values of 0.001 for all metrics. These findings indicate that the observed metabolomic separation between healthy controls and MDS/CMML samples is unlikely to result from random class assignment and is consistent with the presence of a reproducible disease-associated metabolic signature, despite only moderate overall predictive performance.

To address the limited size of the discovery cohort and assess the robustness of our findings, we validated the identified metabolic phenotype in an independent external patient cohort. Despite expected inter-cohort differences in several amino acid (glycine and alanine) and energy metabolites (citrate, glycerol, and acetone), the overall plasma metabolomic profile remained remarkably similar between the cohorts. In particular, the dominant alterations affecting lipoprotein-associated metabolites were preserved, supporting the reproducibility of the disease-associated metabolic phenotype across independent patient populations (Supplementary Figure S3). As the external cohort did not include patients with CMML, the CMML-enriched metabolic alterations could not be independently validated.

## 4. Discussion

In this study, we identified a disease-associated plasma metabolic signature in patients with MDS and CMML, characterized by broad suppression of circulating lipoprotein-associated metabolites and a CMML-enriched increase in ketone body metabolites, particularly 3-HB. Importantly, although ketone body levels were highest in CMML, the direct comparison between MDS and CMML did not reach statistical significance; these alterations should therefore not be interpreted as CMML-specific. Moreover, given the small number of CMML patients in the discovery cohort (n = 8) and the biological heterogeneity of CMML, these observations should be considered exploratory and cannot be assumed to represent a general metabolic feature of CMML. External cohort analysis confirmed the major MDS-associated plasma metabolomic pattern. Unsupervised clustering revealed distinct metabolomic profiles between healthy controls and patients with MDS or CMML, while PLS-DA supported a reproducible, although only moderately predictive, separation between healthy and disease samples. Importantly, 3-HB levels were not associated with mutational burden, blast percentage, hematologic severity, clinical risk groups, or immunophenotypic parameters, suggesting that this metabolic phenotype is not simply a surrogate of disease burden.

The most prominent shared alteration across MDS and CMML was the reduction of multiple lipid- and lipoprotein-associated metabolites, including HDL-, LDL-, IDL-, and apolipoprotein-associated fractions. This included decreased apolipoprotein A-I/-II-associated HDL fractions, HDL3-associated free cholesterol, triglyceride-containing HDL fractions [27], and several LDL phospholipid fractions. ApoA1 is the major structural protein of HDL and plays a central role in reverse cholesterol transport, cellular cholesterol efflux, and anti-inflammatory HDL functions [28]. ApoA2, the second most abundant HDL-associated apolipoprotein, contributes to HDL particle stability, remodeling, and regulation of lipase-dependent lipid metabolism [29]. Therefore, the reduced abundance of ApoA1- and ApoA2-associated fractions suggests altered HDL composition and systemic lipoprotein homeostasis in MDS/CMML. However, plasma metabolomic measurements alone cannot determine whether these changes result from altered lipoprotein production or clearance, increased peripheral lipid utilization, inflammation-associated remodeling, nutritional factors, or medication effects. Functional studies assessing lipoprotein turnover and HDL functionality, including cholesterol efflux capacity, antioxidant activity, and anti-inflammatory effects, will be required to determine the underlying mechanisms.

In addition to reduced ApoA1- and ApoA2-associated HDL fractions, we observed decreased HDL3-associated free cholesterol fractions. Because HDL3 particles are small, dense HDL subclasses involved in cholesterol efflux, antioxidant defense, and reverse cholesterol transport, these changes may indicate altered HDL maturation, remodeling, or cholesterol trafficking [30]. Changes in triglyceride-containing HDL fractions further support altered HDL particle composition and remodeling in MDS/CMML. Together with changes in LDL- and IDL-associated metabolites, these findings suggest a broader disturbance of systemic lipoprotein homeostasis and cholesterol distribution between HDL- and LDL-associated fractions [31] in MDS/CMML. Collectively, the relative enrichment of HDL-associated metabolites in healthy controls and their reduced abundance in MDS and CMML indicate disease-associated alterations in circulating lipoprotein composition and homeostasis, though without establishing the underlying mechanism.

In contrast to the shared suppression of lipoprotein-associated metabolites, CMML samples in our discovery cohort showed a more pronounced increase in ketone body-associated metabolites, including 3-HB, acetoacetate, and succinate. 3-HB is a major circulating ketone body generated primarily from fatty acids and may reflect altered systemic fatty acid utilization, hepatic ketogenesis, nutritional state, or inflammatory catabolic metabolism [32]. Importantly, circulating ketone body concentrations are strongly influenced by fasting duration and recent dietary intake. As these parameters were not standardized or documented in our cohort, we cannot exclude that differences in nutritional state contributed to the higher 3-HB and acetoacetate levels observed in CMML. Notably, elevated 3-HB in CMML should not be interpreted as direct evidence of increased fatty acid oxidation in malignant monocytes. To further characterize lipid metabolism, we performed LC-MS analysis of plasma carnitine species and fatty acids. While free carnitine abundances were comparable across healthy controls, MDS, and CMML, CMML samples exhibited increased levels of C18:1-acylcarnitine, together with elevated oleic and isopalmitic acids. Acylcarnitines are intermediates of the carnitine shuttle that transports long-chain fatty acids into mitochondria for FAO, whereas isopalmitic acid and oleic acid, the most abundant monounsaturated fatty acid, are substrates for this pathway. These findings complement the NMR-derived metabolomic profile and support disease-associated alterations in systemic lipid metabolism and carnitine-dependent fatty acid handling.

Re-analysis of publicly available transcriptomic datasets [25,26] demonstrated increased expression of lipid uptake and trafficking genes, including *FABP5*, *APOE*, *LPL*, and *SLC27A2*, in primary CMML monocytes. This transcriptional pattern is consistent with enhanced expression of pathways related to lipid acquisition and intracellular lipid handling but was not accompanied by coordinated activation of downstream FAO or ketone-utilization pathways. Importantly, gene-expression changes alone do not establish increased functional lipid uptake, intracellular lipid accumulation, or metabolic flux. Together, the plasma metabolomic, LC-MS, and transcriptomic data suggest altered systemic lipid metabolism accompanied by changes in lipid-handling transcriptional programs in CMML. Whether these transcriptional changes translate into a functional lipid-acquisition phenotype remains to be determined.

Although our re-analysis did not reveal induction of FAO genes, recent multi-omic studies have reported enrichment of broader fatty acid metabolism and oxidative phosphorylation signatures in CMML monocytes, together with altered inflammatory and macrophage polarization programs [26]. Given the close biochemical relationship between fatty acid metabolism, ketogenesis, mitochondrial activity, and inflammatory signaling, elevated 3-HB levels in CMML may therefore reflect systemic metabolic adaptation linked to monocytic differentiation and inflammatory lipid remodeling. However, direct functional studies, including FAO flux assays and isotope tracing experiments, will be required to determine whether these alterations reflect increased FAO, impaired fatty acid utilization, or broader inflammatory-catabolic stress.

An intestinal contribution to elevated circulating 3-HB appears less likely based on preliminary fecal microbiome and metabolite data, which did not show enrichment of 3-HB-producing bacteria or increased fecal 3-HB levels in MDSs compared with healthy controls (data available on request). Future studies integrating plasma metabolomics with fecal metabolomics, microbiome profiling, dietary information, and fasting status will be required to determine the relative contribution of host and microbial metabolism to circulating ketone body levels in myeloid neoplasms.

Beyond lipid metabolism, we also observed reduced circulating lysine levels in patients with MDS and CMML. Lysine is an essential amino acid involved in protein synthesis and several processes relevant to myeloid disease biology, including epigenetic regulation, extracellular matrix remodeling, and carnitine biosynthesis. Although the biological significance of reduced plasma lysine in myeloid neoplasms remains unclear [33], it may reflect broader alterations in amino acid metabolism accompanying malignant hematopoiesis. Because free plasma carnitine abundances remained unchanged in our LC-MS analysis, our data do not support reduced carnitine availability as an explanation for the observed lipid metabolic phenotype. Future studies should therefore investigate lysine metabolism independently of systemic carnitine levels, including its potential relationship to epigenetic regulation [34,35] and bone marrow microenvironment remodeling [36].

Several limitations of this study should be considered. First, the CMML subgroup in the discovery cohort was small (n = 8). Given the substantial clinical, molecular, and biological heterogeneity of CMML, this sample size does not allow us to determine whether the observed elevations in ketone bodies and selected lipid-associated metabolites represent a broadly shared feature of CMML or a metabolic phenotype present only in a subset of patients. Although individual data points are presented in Figure 2, larger and molecularly characterized CMML cohorts are required to assess the reproducibility and prevalence of this metabolic phenotype. The independent validation cohort consisted exclusively of MDS patients and therefore validated only the MDS-associated metabolic phenotype, while the CMML-enriched findings could not be independently validated. Second, the time from blood sampling to centrifugation was not documented, which may introduce pre-analytical bias for labile metabolites such as glutamine, glutamate, and lactate. For this reason, glucose and lactate were excluded from the main analyses. Third, fasting status, recent dietary intake, and the time of blood collection were not standardized or documented, all of which may influence circulating lipid, triglyceride, ketone body, and other metabolite levels. This is particularly relevant to the observed differences in 3-HB and acetoacetate, which are highly sensitive to fasting and nutritional state. Consequently, we cannot determine to what extent the observed variation in ketone body levels reflects disease-associated metabolic alterations versus differences in nutritional or fasting status. Fourth, healthy controls and patient groups differed in sex distribution and lipid-lowering medication use, with more females and slightly more hypolipidemic drug use among healthy controls. However, sensitivity analyses adjusting for sex and lipid-lowering medication use showed that the principal ketone body- and lipoprotein-associated differences remained significant, including after FDR correction across the selected metabolite panel. Nevertheless, residual confounding by other factors, particularly fasting and nutritional status, cannot be excluded and should be addressed in future validation cohorts. Finally, the cross-sectional design precluded assessment of the intraindividual stability and temporal reproducibility of the identified metabolic signatures. Longitudinal studies with standardized pre-analytical sampling protocols will be important to determine the robustness and biological stability of these metabolomic profiles over time.

## 5. Conclusions

In conclusion, our study identifies broad alterations in systemic lipid metabolism in MDSs and CMML. While both diseases exhibited broad reductions in circulating lipoprotein-associated metabolites, the CMML subgroup showed more pronounced alterations in ketone bodies and selected acylcarnitines and fatty acids, together with a monocyte transcriptional program consistent with enhanced lipid uptake and intracellular lipid handling. Given the limited CMML sample size, these findings should be considered exploratory and require validation in larger CMML cohorts. Together, these findings support altered systemic lipid metabolism and carnitine-dependent fatty acid handling as metabolic features associated with these myeloid neoplasms, while highlighting the need for future functional studies to determine their mechanistic and biological significance.

## Figures and Tables

**Table 1:** Demographic and clinical characteristics of the discovery cohort.

	Healthy Controls (n = 14)	MDS (n = 22)	CMML (n = 8)
Age, years, median (range)	60 (54–70)	66.5 (43–81)	67.5 (41–72)
Sex, male/female, n	6/8	19/3	5/3
BMI, kg/m^2^, median (range)	25.6 (22.9–37.0)	27.1 (19.0–36.7)	25.0 (19.6–30.4)
Cardiovascular comorbidity, n (%)	8 (57%)	14 (64%)	6 (75%)
Lipid-lowering medication, n (%)	4 (29%)	2 (9%)	3 (38%)
Lower-risk disease, n (%)	NA	10 (45%)	5 (63%)
Higher-risk disease, n (%)	NA	12 (55%)	3 (38%)

## Data Availability

The raw data supporting the conclusions of this article will be made available by the authors on request.

## Supplementary Materials

The following supporting information can be downloaded at: https://www.mdpi.com/article/10.3390/metabo16090648/s1, Table S1: Flow cytometry antibody panel; Table S2: Sensitivity analysis of selected metabolites adjusted for sex and lipid-lowering medication use; Figure S1: LC-MS analysis of selected plasma carnitine and fatty-acid features; Figure S2: Performance of PLS-DA models with increasing numbers of latent components; Figure S3: Validation of the plasma metabolomic phenotype in an independent external cohort.
