# Correction: Balaian et al. Systemic Metabolic Changes in Plasma of Patients with Myelodysplastic Neoplasms and Chronic Myelomonocytic Leukemia. *Metabolites* 2026, *16*, 648

**DOI:** 10.3390/metabo16100730

**Published:** 2026-09-29

**Authors:** Ekaterina Balaian, Iryna Kovtun, Fabian Springer, Denise Medeiros Selegato, Sophie Jonas, Uta Oelschlaegel, Manja Wobus, Michael Wulfert, Corinna Strupp, Ulrich Germing, Michael Zimmermann, Martin Bornhäuser, Triantafyllos Chavakis, Katja Sockel, Alexander Funk

**Affiliations:** 1 Department of Internal Medicine I, University Hospital Carl Gustav Carus Dresden, Dresden University of Technology, 01307 Dresden, Germany; iryna.kovtun@ukdd.de (I.K.); uta.oelschlaegel@ukdd.de (U.O.); manja.wobus@ukdd.de (M.W.); martin.bornhaeuser@ukdd.de (M.B.); katja.sockel@ukdd.de (K.S.); 2 German Cancer Consortium (DKTK), Partner Site Dresden and German Cancer Research Center (DKFZ), 69120 Heidelberg, Germany; 3 National Center for Tumor Diseases (NCT/UCC), 01307 Dresden, Germany; sophie.jonas@ukdd.de (S.J.); triantafyllos.chavakis@ukdd.de (T.C.); alexander.funk@ukdd.de (A.F.); 4 Molecular Systems Biology Unit, European Molecular Biology Laboratory (EMBL), 69117 Heidelberg, Germanydenise.selegato@embl.de (D.M.S.); michael.zimmermann@embl.de (M.Z.); 5 Institute for Clinical Chemistry and Laboratory Medicine, University Hospital Carl Gustav Carus, Dresden University of Technology, 01307 Dresden, Germany; 6 Department of Hematology, Oncology, and Clinical Immunology, Heinrich-Heine University, 40225 Dusseldorf, Germany; michael.wulfert@med.uni-duesseldorf.de (M.W.); corinna.strupp@med.uni-duesseldorf.de (C.S.); germing@med.uni-duesseldorf.de (U.G.)

The authors would like to make the following correction to their published paper [1]. The change is as follows:

**Back Matter Correction**

We noticed that the Institutional Review Board (IRB) Statement and Informed Consent Statement (ICS) sections were missing from the Back Matter of the article. These sections have now been added.

**Institutional Review Board Statement:** The study was approved by the Ethics Committee of Ethikkommission an der TU Dresden (Approval No: EK289112008, Approval Date: 21 January 2019).
**Informed Consent Statement:** Informed consent was obtained from all subjects involved in the study. Written informed consent has been obtained from the patients to publish this paper.
