# Supplementary material for: Systemic Metabolic Changes in Plasma of Patients with Myelodysplastic Neoplasms and Chronic Myelomonocytic Leukemia
Source: Metabolites. 2026 Sep 4;16(9):648. doi: 10.3390/metabo16090648 (PMC13608964; doi:10.3390/metabo16090648)
Supplement: Supplementary file 1 [file metabolites-16-00648-s001.zip › metabolites-4503465-supplementary.pdf]

Supplementary material

Supplementary Table S1. Flow cytometry antibody panel.

	FITC	PE	PC5.5	PE-Cy7	Alexa F 647	APC AF700	APC AF750	PB	KrO
**Treg**	**CD45RA**	**CD25**	**CD39**	**CD4**	**FoxP3**	**CD127**	**CD3**	**Helios**	**CD45**
	DuraClone IM Treg B53346 (BC) and CD127 (BC)								
**T lymphocytes**	**CCR-7 (CD197)**<br>BD 561271	**CD45RA**<br>BD 555489	**CD3**	**CD28**<br>BD 560684	**CD27**<br>BD 558664		**CD8**<br>BD 641400	**CD4**	**CD45**
**NK cells**	**CD16**<br>BC B49215	**KIR2DL2**<br>R&D FAB1848P	**CD56**<br>eBio35-0567-42	**KIR2DL1**<br>eBio25-1589-42	**NKG2D**<br>BioLeg320808		**CD3**<br>BD 641415	**KIR3DL1**<br>BLD 312714	**CD45**
**NK cells**	**DNAM1**<br>BLD 338304	**NKp30**<br>BLD 325208	**CD56**<br>eBio35-0567-42	**CD16**<br>BD335823	**NKp46**<br>BLD 331918		**CD3**<br>BD 641415		**CD45**

BC - Beckman Coulter; BD - Becton Dickinson; R&D - R&D Systems Antibodies; eBio- eBioscience Thermo Fisher Scientific; BLD – Biolegend

Supplementary Table S2. Sensitivity analysis of selected metabolites adjusted for sex and lipid-lowering medication use.

Metabolite	MDS vs healthy p	MDS vs healthy FDR q	CMML vs healthy p	CMML vs healthy FDR q	CMML vs MDS p	CMML vs MDS FDR q
3-HB	0,1990	0,2874	0,0051	**0,0156**	0,0700	0,2275
Acetoacetate	0,6020	0,6450	0,0138	**0,0256**	0,0290	0,1907
Succinate	0,6450	0,6450	0,0191	**0,0310**	0,0390	0,1907
ApoA1-HDL	0,0000	**0,0002**	0,0063	**0,0156**	0,7650	0,7650
ApoA2-HDL	0,0002	**0,0003**	0,1100	0,1430	0,5380	0,7046
ApoB-LDL	0,0001	**0,0003**	0,0020	**0,0130**	0,5420	0,7046
ApoB-IDL	0,0001	**0,0003**	0,9540	0,9540	0,0440	0,1907
CHOL-LDL1	0,0007	**0,0012**	0,0071	**0,0156**	0,2170	0,5330
CHOL-LDL2	0,5210	0,6157	0,8040	0,8710	0,6990	0,7573
fCHOL-HDL3	0,0001	**0,0003**	0,0310	**0,0448**	0,4950	0,7046
PHOSL-LDL1	0,0000	0,0000	0,0011	**0,0130**	0,3580	0,6649
PHOSL-LDL2	0,2710	0,3523	0,5810	0,6866	0,6910	0,7573
PHOSL-LDL4	0,0001	**0,0003**	0,0072	**0,0156**	0,2460	0,5330

Multivariable regression analysis was performed for selected metabolites representing the principal findings of the study. Models were adjusted for sex and lipid-lowering medication use. Metabolite concentrations were log-transformed before analysis. Nominal p values and FDR-adjusted q values are shown. Bold values indicate statistical significance after Benjamini–Hochberg FDR correction ($q < 0.05$)

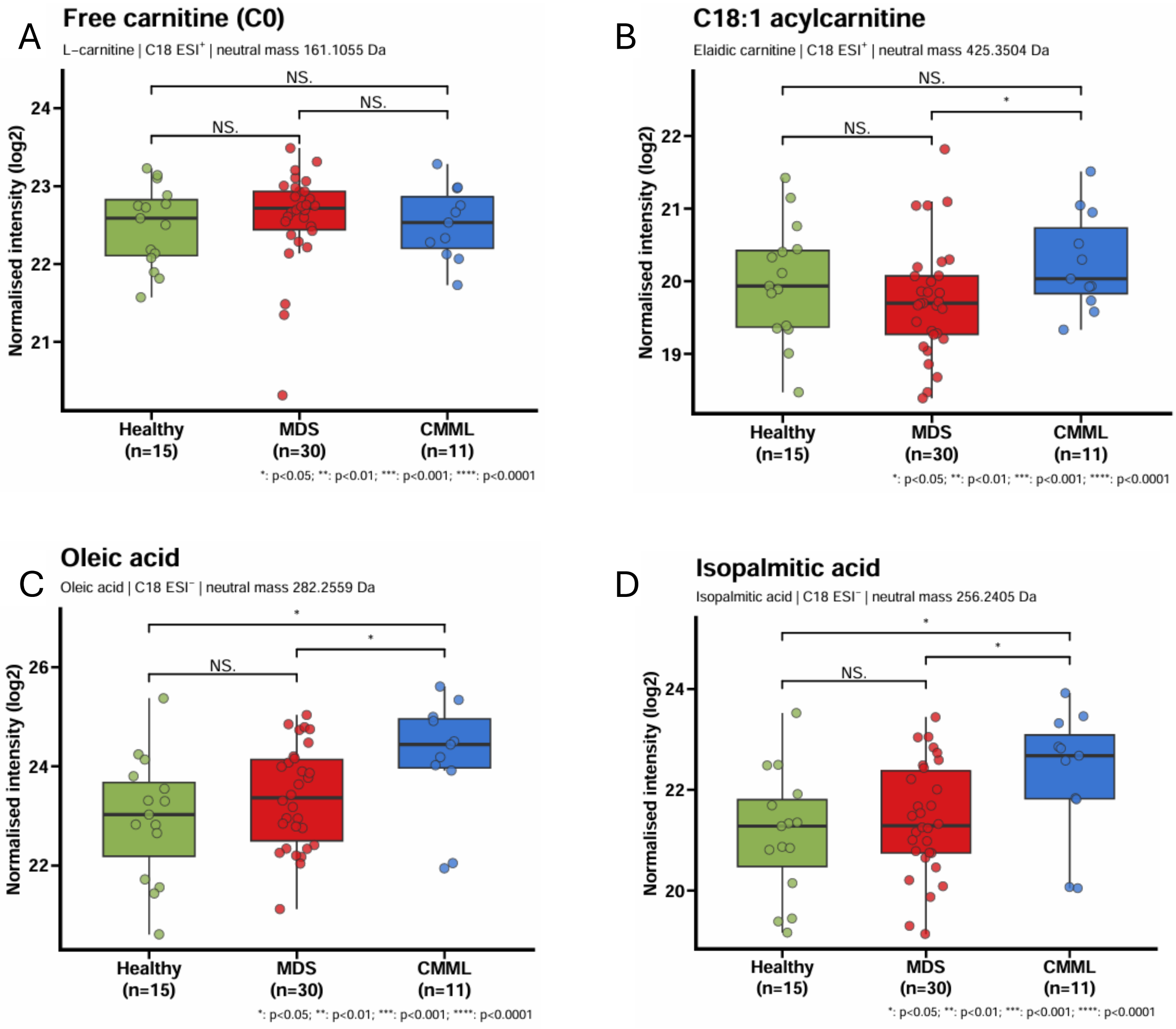

Supplementary Figure S1. LC-MS analysis of selected plasma carnitine and fatty-acid features. Normalized abundances of (A) free carnitine, (B) C18:1 acylcarnitine, (C) oleic acid, and (D) isopalmitic acid are shown for healthy controls and patients with MDS or CMML. Free carnitine and C18:1 acylcarnitine were putatively annotated by MS1 accurate-mass matching against METLIN; the geometric or positional isomer identity of the C18:1 acylcarnitine could not be resolved. Oleic acid and isopalmitic acid were annotated by accurate-mass and retention-time matching against an in-house reference library acquired using the same chromatographic column. Statistical comparisons were performed using two-sided Wilcoxon rank-sum tests. * $p<0.05$; NS, not significant.

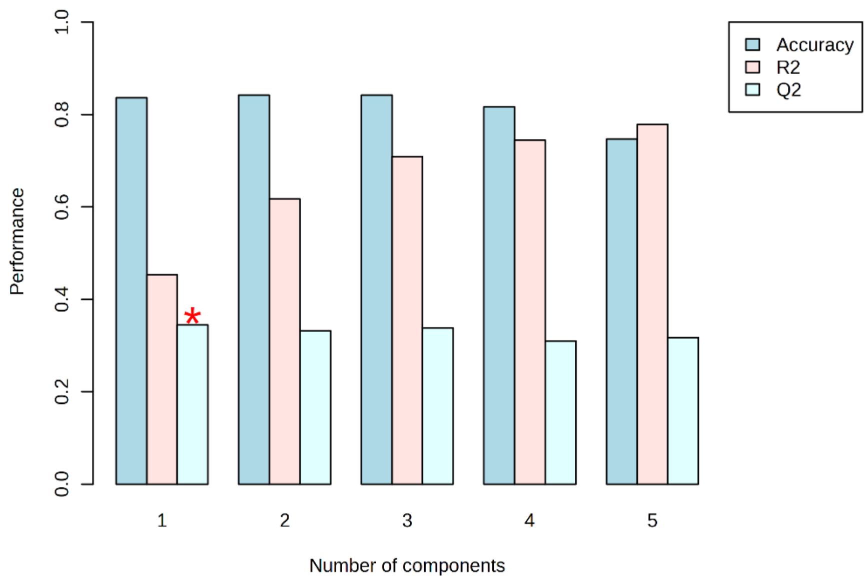

Supplementary Figure S2. Performance of PLS-DA models with increasing numbers of latent components.

Cross-validated accuracy, R^2, and Q^2 are shown for models containing one to five latent components. The one-component model (red asterisk) was selected for subsequent analyses.

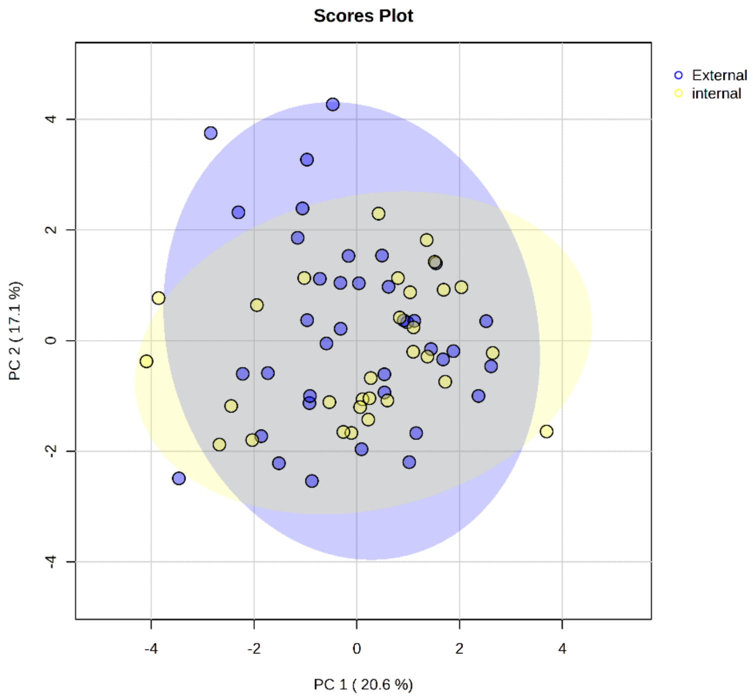

Supplementary Figure S3. Validation of the plasma metabolomic phenotype in an independent external cohort.

Principal component analysis (PCA) scores plot comparing the internal discovery cohort (yellow) and the independent external validation cohort (blue). Despite expected inter-cohort variability, substantial overlap of the overall metabolomic profiles was observed, indicating comparable global metabolic signatures.
